# Hyperactivity and Euphoria Without Agitation Due to Hyoscyamus niger (Henbane) Poisoning in a Child

**DOI:** 10.7759/cureus.107588

**Published:** 2026-04-23

**Authors:** Aykut Yucal, Melike Yucal

**Affiliations:** 1 Emergency Medicine, Harakani State Hospital, Kars, TUR; 2 Pediatrics, Harakani State Hospital, Kars, TUR

**Keywords:** anticholinergic toxicity, emergency medicine, euphoria, henbane poisoning, hyoscyamus niger, hyperactivity, pediatric toxicology

## Abstract

*Hyoscyamus niger* (henbane) is a toxic plant containing tropane alkaloids that can cause anticholinergic toxicity. Pediatric presentations may differ from adults and may include atypical behavioral manifestations such as hyperactivity and euphoria without agitation. An 11-year-old previously healthy boy presented with sudden behavioral changes, hyperactivity, and unexplained euphoria. Approximately four hours earlier, he had ingested about one tablespoon of fresh *Hyoscyamus niger* seeds. Physical examination revealed minimal mydriasis and mild flushing. The patient was cooperative but disoriented, with visual hallucinations. Laboratory tests were normal, and electrocardiography showed sinus tachycardia. Findings were consistent with anticholinergic toxicity. Activated charcoal and physostigmine were not administered due to delayed presentation and absence of severe or life-threatening symptoms. Symptoms resolved completely within 24 hours with supportive care. In conclusion, *Hyoscyamus niger* intoxication may present predominantly with hyperactivity and euphoria without agitation, representing an atypical behavioral phenotype in pediatric patients. Plant-derived toxicities should be considered when acute behavioral changes occur, especially in rural settings.

## Introduction

*Hyoscyamus niger*, known in the regions where it grows in Turkey as “bengildek,” “gavur haşhaşı,” “banotu,” “kara banotu,” “deli bat bat otu,” in Turkish and by its English name “henbane,” is a plant that has attracted attention throughout history for both its medicinal and toxic effects. The name “henbane” originates from Medieval English and means “chicken poison/plant that kills chickens”; this name is thought to be related to the behavioral changes and mortality it causes in animals [1].

Morphologically, *Hyoscyamus niger* is characterized by hairy stems, broad, irregular leaves, and bell-shaped flowers with dark purple venation. Its capsule-shaped fruits contain numerous small seeds. Historically, Hyoscyamus species have been used for their analgesic, sedative, and hallucinogenic effects [2,3].

The plant's pharmacological and toxic properties are based on its high levels of tropane alkaloids, particularly hyoscyamine, atropine, and scopolamine. These alkaloids exert a potent anticholinergic effect via muscarinic receptor antagonism and, in cases of poisoning, lead to typical anticholinergic toxicosis symptoms such as mydriasis, tachycardia, hot-dry skin, dry mouth, agitation, delirium, and hallucinations [3,4]. Recent phytochemical studies have shown that alkaloid content can vary significantly depending on the geographical and environmental conditions in which the plant grows; this suggests that clinical severity may vary even with the same amount of consumption [5].

Poisonings associated with *Hyoscyamus niger* occur mainly in children and in rural areas as a result of accidental consumption of the plant. In addition, there are examples of use with the expectation of a narcotic effect [6]. Anticholinergic symptoms in patients present in a wide variety of ways; there are reported cases where recovery was achieved without serious complications with appropriate supportive treatment, but there are also cases requiring the use of physostigmine, a specific antidote, due to severe central nervous system findings, severe agitation, or cardiac effects [7,8].

Increased ethnobotanical interest in recent years, regional variability in content, and new case reports have brought *Hyoscyamus niger* back into focus in modern clinical toxicology. A thorough understanding of both its traditional uses and its potential toxicity is essential for accurate diagnosis and treatment, particularly in rural areas [4,5].

This case report presents a pediatric patient who presented following oral intake of *Hyoscyamus niger* in the province of Kars, Turkey, one of the regions where the plant is found. In addition to classic anticholinergic toxicity findings, the clinical course was dominated by behavioral changes characterized by marked hyperactivity and unexplained euphoria. Although agitation, confusion, and delirium are frequently described in *Hyoscyamus niger* poisoning, this case is notable for a predominantly euphoric and hyperactive presentation without agitation, which represents an atypical clinical pattern in the pediatric population. This case highlights the importance of recognizing this atypical behavioral presentation for differential diagnosis and clinical management; it aims to increase awareness among emergency department physicians, particularly in rural areas, regarding similar cases that may be encountered.

## Case presentation

**Figure 1:**
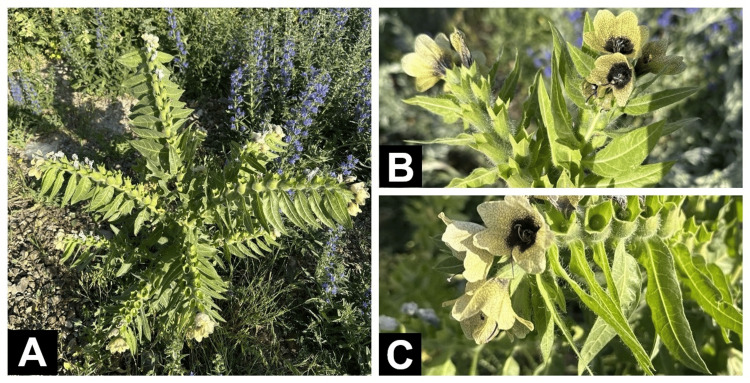
Images of the Hyoscyamus niger (henbane) plant taken by our authors around the Susuz district in Kars province, Turkey A: Whole-plant habit showing erect growth and densely pubescent, lobed leaves B: Inflorescence with pale yellow corolla, dark venation, and central purple throat C: Close-up of flowers and developing capsules with prominent glandular hairs

An 11-year-old male patient with no known chronic illness was brought to the emergency department by his family due to sudden behavioral changes, hyperactivity, and unexplained euphoria. The patient's family reported that approximately four hours before presentation, the patient had taken about one and a half tablespoons of unripe white seeds from the fruit of a plant known locally as “bat bat otu” (*Hyoscyamus niger*) orally. The family became suspicious when they noticed that the child's behavior resembled that of someone who had consumed alcohol, and they sought emergency department after learning about the plant intake. There was no history of vomiting or other gastrointestinal symptoms. The plant was identified by comparing the family's knowledge of its local name with plant images found in the literature (Figure 1).

Upon admission to the emergency department, the patient was cooperative but significantly hyperactive and excessively euphoric; inappropriate jokes were made, and attention was easily distracted. Person and situational orientation were impaired, but consciousness was clear, and the Glasgow Coma Scale score was assessed as 15 [9]. Visual hallucinations were present; the patient stated that he perceived the bed environment as a car. Agitation, aggression, or loss of consciousness was not observed.

Vital signs were as follows: temperature: 37.3°C, blood pressure: 128/83 mmHg, pulse: 120-130/min, respiratory rate: 17/min, and oxygen saturation: 99%. Physical examination revealed minimal midriasis of the pupils. The skin was moist, with mild flushing, particularly on the face; no dry mouth was noted. Intestinal sounds were normal, and urine output was normal. No focal deficit was observed on neurological examination. No additional pathological findings were noted on systematic examination.

No additional pathological findings were detected on the electrocardiogram other than sinus tachycardia. Laboratory tests showed that complete blood count and biochemical parameters were within normal limits (Table 1). Based on the clinical picture, history, and examination findings, a preliminary diagnosis of anticholinergic toxicity due to *Hyoscyamus niger* intake was considered. Although toxicological confirmation was not available, the diagnosis was considered clinically plausible based on the characteristic findings and exposure history. 

**Table 1:** The laboratory results of the patient's emergency department admission ALP: Alkaline phosphatase; ALT: Alanine aminotransferase; aPTT: Activated partial thromboplastin time; AST: Aspartate aminotransferase; CRP: C-reactive protein; GGT: Gamma-glutamyl transferase; INR: International normalized ratio; LDH: Lactate dehydrogenase; PT: Prothrombin time; WBC: White blood cell.

Parameter	Result	Reference range	Interpretation
WBC	8.6 × 10³/µL	4.5-13	Normal
Neutrophils	56.6%	–	Normal
Lymphocytes	31.6%	–	Normal
Hemoglobin	12.9 g/dL	12-16	Normal
Platelets	282 × 10³/µL	150-450	Normal
CRP	4.6 mg/L	<5	Normal
Troponin T	3 ng/L	<14	Normal
Glucose	92 mg/dL	70-140	Normal
Urea	25.7 mg/dL	16.6-48.5	Normal
Creatinine	0.63 mg/dL	0.4-0.8	Normal
Sodium	137 mmol/L	135-145	Normal
Potassium	4.2 mmol/L	3.5-5.0	Normal
Calcium	9.2 mg/dL	8.5-10.5	Normal
ALT	15 U/L	0-41	Normal
AST	23 U/L	0-40	Normal
GGT	9 U/L	8-61	Normal
ALP	103 U/L	40-130	Normal
LDH	196 U/L	135-280	Normal
pH	7.39	7.35-7.45	Normal
pCO₂	42.3 mmHg	35-45	Normal
HCO₃⁻	24.0 mmol/L	22-26	Normal
Base excess	–0.1	–2 to +2	Normal
SaO₂	97.9%	95%-100%	Normal
PT	14 sec	11.9-17.0	Normal
INR	1.0	0.8-1.2	Normal
aPTT	29.4 sec	20.0-38.0	Normal

Activated charcoal was not administered because more than four hours had passed since the oral intake of the plant, and the patient exhibited central nervous system findings that could pose a significant aspiration risk. The patient was observed under intravenous hydration and close cardiac and neurological monitoring. Intravenous midazolam was kept on standby in case of possible seizure or severe agitation, but its use was not required. Physostigmine treatment was not administered due to the absence of life-threatening or refractory anticholinergic symptoms.

During the 24-hour observation period, the patient's hallucinations and behavioral symptoms gradually subsided. Control laboratory tests remained within normal limits. Upon complete resolution of clinical findings, the patient was discharged after a one-day hospitalization without any additional neurological sequelae. Written informed consent was obtained from patient’s parents for publication of this case report.

## Discussion

*Hyoscyamus niger* is a toxic plant with a narrow therapeutic range that produces central and peripheral anticholinergic effects due to the tropane alkaloids it contains. The most commonly reported clinical picture includes classical anticholinergic toxicosis findings such as mydriasis, tachycardia, agitation, confusion, and delirium, whereas atypical presentations, particularly in pediatric patients, may be dominated by behavioral changes such as hyperactivity and euphoria without agitation. Although central nervous system manifestations are well described in the literature, previously reported pediatric cases have more commonly emphasized agitation, confusion, and delirium rather than a predominantly euphoric behavioral pattern, which appears to be less well characterized in the existing pediatric literature [1,4,6-8]. In contrast, our patient presented with marked hyperactivity and inappropriate cheerfulness without agitation or aggression, highlighting a presentation in which hyperactivity and euphoria are more prominent than the classical agitated pattern described in pediatric anticholinergic toxicity.

In the differential diagnosis, other toxic ingestions, substance exposure, delirium-related conditions, and primary neurological disorders were considered. However, the absence of focal neurological deficits, normal laboratory findings, and the clear temporal relationship between plant ingestion and symptom onset supported a toxic etiology. In addition, the presence of characteristic features such as mydriasis, tachycardia, hallucinations, and behavioral changes was consistent with anticholinergic toxicity, making alternative diagnoses less likely.

The edible part of the plant and its physiological state are among the important factors affecting the severity of toxicity. In the presented case, the patient's consumption of fresh, white-colored seeds that had not yet dried out may be a factor influencing the clinical presentation. Previous phytochemical studies have shown that tropane alkaloid concentration can vary depending on the plant's developmental stage, environmental conditions, and harvest time [5,10]. This variability can lead to unpredictable clinical symptoms even with similar amounts consumed. Further studies are needed to better characterize the variability of clinical presentations in pediatric *Hyoscyamus niger* intoxication, particularly regarding atypical behavioral manifestations.

Anticholinergic toxicity does not always present with the classic “dry skin-dry mucous membranes” pattern; atypical presentations may occur, particularly in the pediatric population, as emphasized in the literature [7]. In this case, the moist skin and absence of dry mouth suggest a partial or early form of anticholinergic poisoning. This situation demonstrates that the diagnosis should not be based solely on individual physical examination findings.

In our case, activated charcoal was not administered, and physostigmine was not required. This conservative approach was based on the patient’s stable clinical condition and the absence of severe central or peripheral anticholinergic manifestations requiring specific antidotal therapy. In addition, the fact that more than four hours had passed between ingestion and presentation, together with the potential aspiration risk due to central nervous system findings, further precluded the use of activated charcoal. Furthermore, physostigmine was not indicated because the patient did not present with life-threatening agitation, refractory delirium, or severe cardiac arrhythmia. Complete resolution of clinical findings with supportive treatment and close monitoring is consistent with cases reported in the literature [4,7].

A limitation of this case is the absence of laboratory toxicological confirmation, and the diagnosis was based on clinical findings and history. Additionally, plant identification relied on the family’s report and comparison with images in the literature, which may introduce a degree of uncertainty. However, the characteristic clinical presentation and temporal relationship between ingestion and symptom onset strongly support the diagnosis of *Hyoscyamus niger* intoxication.

## Conclusions

This case highlights that *Hyoscyamus niger* intoxication in pediatric patients may present with hyperactivity and euphoria without agitation, representing an atypical behavioral pattern. In children presenting with acute and unexplained behavioral changes, particularly in rural settings, plant-derived toxicities should be considered in the differential diagnosis.
